# Creating a model of cross-task motivation — A meta-narrative review of the literature on dynamic motivation

**DOI:** 10.3389/fpsyg.2023.1193186

**Published:** 2023-06-16

**Authors:** Frida K. Feyer

**Affiliations:** Department of Leadership and Organizational Behaviour, BI Norwegian Business School, Oslo, Norway

**Keywords:** situational motivation, dynamic motivation, self-determination theory, cross-task, meaningfulness, intrinsic motivation

## Abstract

**Introduction:**

Substantial research on job motivation over the years has identified motivation to be essential to work outcomes such as wellbeing, attitudes, and performance. Yet, research on job motivation addressing temporal influences has been sparse. Existing research has addressed job motivation as an aggregation of the motivation for tasks, ignoring the possibility of temporal effects where the motivation for one task affects motivation in a subsequent task. The current meta-narrative review analyzes existing research on task motivation and synthesizes findings into a model of cross-task motivation.

**Methods:**

Using a predetermined search strategy, a systematic search yielded 1,635 documents of which 17 were selected. Papers were analyzed using a meta-narrative approach according to RAMSES publication standards.

**Results:**

Four key meta-narratives were identified, contributing information from different research traditions; (1) restoration effects after need frustration, (2) intrinsic and extrinsic motivation, (3) cognitive carryover effects, and (4) meaning of work. Synthesizing findings from these meta-narratives, a meta-theoretical model for understanding cross-task motivation was proposed.

**Discussion:**

This model provides an extension of existing motivational theories elucidating temporal motivational processes. Implications for practitioners include the possibility of arranging jobs to maximize positive motivational outcomes.

## 1. Introduction

Job motivation has been extensively studied over the years, as it is crucial to numerous outcomes within the workplace, e.g., employee behavior, job performance, employee wellbeing, and attitudes (Gagné and Deci, 2005; van den Broeck et al., 2016; van Iddekinge et al., 2018; Fishbach and Woolley, 2022). However, little attention has been given to the temporal aspects of job motivation, as motivation within the work domain traditionally has been studied as a fixed contextual phenomenon. Based on the definition of the job as “an aggregation of tasks assigned to a worker” (Wong and Campion, 1991, p. 825), the conceptualization of job motivation has generally entailed an aggregation of different tasks into motivation for the job as a whole. Therefore, there are substantial gaps in the knowledge concerning fluctuations of motivation through the workday and how employee motivation can be optimized from a temporal perspective (Oldham and Hackman, 2010; Deci et al., 2017).

In the modern work environment, employees have jobs consisting of multiple tasks that they have to balance (Ilgen and Hollenbeck, 1991; Raziq and Maulabakhsh, 2015). From the average job consisting of five to six core tasks 30 years ago (Wong and Campion, 1991), an average of 21 tasks per occupation was registered in the O^*^NET Database as of 2019 (National Center for O^*^NET Development, 2019). In addition to this, the workplace has become increasingly complex, with more diverse dimensions (Man and Lam, 2003). In this environment, where employees must portion out resources between multiple, differing tasks, how does motivation vary through the workday?

A review of the past 100 years of motivation research (Kanfer et al., 2017) points to temporal dynamics of motivation as an important future area of research, referencing Roe (2014) in that motivation likely varies as a function of tasks and cycle time. Research into basic psychological need fulfillment that predicts motivation, and motivational processes in general, over the past 20 years, have shown that these concepts do not vary solely between individuals; there is also an in-person day-to-day variation. Reis et al. (2000) found that the need satisfaction of the basic needs of autonomy, competence, and relatedness vary on a day-to-day basis, while van Hooff and van Hooft (2017) found similar results for motivational processes.

Before the year 2022, no organizational studies, to this author's knowledge, had consistently measured within-person day-to-day variation in motivation from the perspective of self-determination theory, despite SDT providing a dynamic perspective on the quantities and qualities of motivation (Gagné et al., 2015), consistent with the conceptualization of motivation as dynamic and ever-changing. As of the fall of 2022, two studies have investigated how measures of within-person variation in motivation and repeated measures of situational (job episode or task level) motivation relate to important outcomes of motivation, such as performance, job satisfaction, and wellbeing, compared to traditional between-person and job level measures. In a diary study of ecological momentary assessment over 30 working days measuring autonomous and controlled motivation, in addition to job satisfaction and productivity, Hogenelst et al. (2022) found that results on the within-person level were more nuanced and did not align with between-person findings from previous studies. At the level of within-persons, motivation measured repeatedly at the task level was not associated with same-day productivity or job satisfaction, contrary to previous findings at the between-persons level. Productivity at the end of the day was negatively associated with next-day task motivation; both controlled and autonomous, while end-of-day job satisfaction was related to next-day controlled task motivation, but unrelated to next-day autonomous task motivation. In a related study, Wang and Panaccio (2022) did multi-level structural equation modeling on data from 158 participants who reported on the need-supportiveness, situational motivation, vitality, and affect of work episodes throughout the workday. In addition, they measured motivation and basic need satisfaction at the job level. They found that while positive correlations between controlled and autonomous motivation existed on a job level, these correlations were not found on the situational level, indicating that situational motivation reacts differently from job motivation, supporting the perspective of the dynamic nature of motivation. The remaining question is then; *how* does situational motivation behave differently from job motivation? The current study seek to address this question by examining the existing literature on changes in motivation across tasks in different fields of research through the method of meta-narrative review. The findings can then be applied to the organizational setting and inform practitioners so that they can be more calibrated and pointed when designing jobs in order to maximize motivational outcomes.

## 2. Theoretical background

### 2.1. The concept of task motivation

The job design literature was early on concerned with designing motivating work, e.g., through the influential work by Herzberg (1968) or by Hackman and Oldham (1974, 1976). The *Job Characteristics Model* (JCM) (Hackman and Oldham, 1974) which is still used to evaluate job motivation today, is based on core job dimensions synthesized from task attributes. Due to this, research on motivational job design tends to not separate motivational tasks from motivational jobs, and so researchers have focused on motivation for jobs as a whole (Wong and Campion, 1991). This measure of motivational jobs would entail the aggregation of motivation for multiple tasks, such as either the aggregation in the JCM or a construct of job motivation. Job motivation is conceptually different from task motivation, as task motivation is the situational experience of motivation for the given task or work situation. In their study from 1991, Wong and Campion investigated how the motivational value of jobs can be predicted from the motivational value of tasks, task interdependence, and task similarity. They tested their model for predicting motivational value on 67 jobs and found that the motivational value of tasks in a job correlated only moderately with the motivational value of jobs, indicating that although there is a positive relationship between motivation for tasks and motivation for jobs, job motivation is not simply an aggregation of task motivation. These results are in line with the findings of Hogenelst et al. (2022) and Wang and Panaccio (2022), illustrating the situational quality of motivation toward different tasks within a job.

### 2.2. Self-determination theory and task motivation

One of the most influential theories of human motivation is the *Self-Determination Theory* (SDT) by Deci and Ryan (1985). The theory is a macro theory of motivation that differentiates between types of motivation that have different catalyzers and consequences, namely autonomous and controlled motivation, and intrinsic and extrinsic motivation (Deci et al., 2017). Autonomous and controlled motivation refers to either engaging in an activity out of free will and by choice or because a power dynamic or contingent reward directs you to. Intrinsic motivation is here a type of autonomous motivation, while extrinsic motivation can be either controlled or autonomous, depending on how attaining the extrinsic consequence is perceived (is it in concurrence with values, is it self-controlled, etc.). Motivation such as job motivation is predicted through how social context support or frustrates the fulfillment of three basic psychological needs; the need for autonomy, relatedness, and competence, with the fulfillment of needs predicting autonomous motivation, and specifically intrinsic motivation (Deci and Ryan, 2000).

Studies of motivation within SDT have been applied across multiple domains and have become central to studies of multiple job outcomes, but these studies have traditionally not differentiated clearly between catalyzers and consequences of motivation at different levels, e.g., the task and job level. SDT does contain the notion that motivational processes may operate on a more general, trait-like level of *causality orientations* (Deci and Ryan, 1985) and a more domain-specific level, *regulatory styles* (Ryan and Connell, 1989), but neither of these concepts captures the state-like level of motivation that takes place on a situational level, e.g., the task level.

A perspective on the SDT that seeks to expand upon these different levels of motivation, is the *Hierarchical Model of Intrinsic and Extrinsic Motivation* (Vallerand, 1997, 2001). This model builds on SDT, utilizing the concepts of intrinsic and extrinsic motivation, and differentiates between three levels of motivation: the global (personality) level, the contextual (life domain) level, and the situational (task) level. Motivation on these three levels of generality interplay through bottom-up, top-down, and horizontal effects, so that motivation on one level may affect motivation on another level (top-down and bottom-up effects), or that motivation in one context may affect motivation in another context (horizontal effects) (Vallerand, 2001). The model has been tested in sport psychology and educational contexts (Lavigne et al., 2009; Lavigne and Vallerand, 2010; Núnez and León, 2018), and in the study of Wang and Panaccio (2022), but evidence in the organizational context is still sparse. In the educational context, motivation toward science courses (contextual level) was predicted by repeated changes in motivation toward science-related activities (task level) (Lavigne and Vallerand, 2010). This illustrates how the model contributes an alternative to the thought that job motivation is an aggregate of task motivation, where change in motivation between tasks (cross-task motivation) is as central as the mere sum of task motivation, and so “the whole is other than the sum of its parts” (Koffka in Heider, 1977, p. 383).

## 3. The current study

The current meta-narrative review tries to answer the question of *how* motivation on the situational level of tasks seem to behave differently from motivation on the job level, by identifying and analyzing the research that has examined task-specific motivational processes of cross-task motivation. This, in order to propose a synthesis of the findings, outlines a possible model of the mechanisms of how motivation for one task affects motivation for another, thereby explaining the dynamic nature of motivation on the task level.

Such a model would contribute to existing theories of motivation in several ways. Firstly, it would expand upon the temporal understanding of motivation in accordance with calls for research on temporal changes in motivation (e.g., Shipp and Cole, 2015; Kanfer et al., 2017) as well as provide a foundation for further investigations of motivation using with-in subject designs. In addition, the model would expand upon SDT and the hierarchical model of Vallerand (1997, 2001) by outlining how cross-task motivation illuminates these temporal aspects of motivation. It is somewhat commonsensical that people are more motivated at some times than others, but the previous studies of Hogenelst et al. (2022) and Wang and Panaccio (2022) display how this still is largely overlooked as influential in the motivation literature.

Knowledge of how different tasks in a job may elicit different levels and types of motivation, and how these differences between tasks may interplay, would enable practitioners to organize work in ways that maximize favorable outcomes of motivation and minimize unfavorable outcomes. This knowledge would also inform practitioners and researchers in other areas, e.g., when ordering effects in surveys, to maximize motivation in participants.

As shown in the preceding sections, job motivation has been researched in a number of different organizational topic areas, and under different research traditions. In order to encompass all contributions to further understanding cross-task motivation, and building sound theory based on all available research, a meta-narrative approach is utilized in the current study.

## 4. Method

The literature review has an important position within organizational psychology, as the field is continuously growing and expanding. However, arguments have been made that traditional literature reviews lack rigor and replicability and that even when rigor is maintained, the chosen methodology may not match the content of the material or the purpose of the contribution (Tranfield et al., 2003; Snyder, 2019). While systematic literature reviews generally are useful for reporting on the effect size and quality of findings in fields of research with relatively homogenous methodology across studies, reviews of heterogeneous topic areas where researchers differ in their conceptualizations and methodologies have presented a challenge (Greenhalgh et al., 2005). The current review looks at the research into cross-task motivation, and how previous motivational experiences in one task may affect motivation in a subsequent task. As most research looking at motivation has done so at the contextual level, rather than the task level, the studies for inclusion in the current review are somewhat scattered and heterogeneous across research fields. Therefore, the present review was conducted using meta-narrative review methods. The meta-narrative review method was first developed by Greenhalgh et al. (2005) and has been defined as a “relatively new method of systematic review, designed for topics that have been differently conceptualized and studied by different groups of researchers” (Wong et al., 2013, p. 2). Based on the guidelines provided by Greenhalgh et al. (2005), the RAMESES publication standards (Wong et al., 2013), and the PRISMA statement (Moher et al., 2009) the present meta-narrative review was conducted on the task level research on motivation across tasks, using meta-narrative methods. In the current study, this entailed a comprehensive search in relevant databases across topic areas, in order to ensure, as far as possible, that all relevant contributions were included. A rigorous screening phase was then carried out where a large number of initial inclusions were examined and compared with the inclusion and exclusion criteria before a second screening phase conducted by two reviewers established the final sample. This sample was analyzed in accordance with the research questions and synthesized in order to meta-theorize about the mechanisms of cross-task motivation.

The current review has been carried out in concordance with the guiding principles of meta-narrative reviews (Wong et al., 2013). This was done by including research from different research traditions and judging the quality of the studies by rules intrinsic to their tradition, and by seeking to synthesize higher-order insight from similarities and differences between research traditions. A meta-narrative review will always be swayed by the research tradition of which the researcher originates, and so this review defines motivation in line with the definition of work motivation by Pinder (2008, p. 11), that work motivation is set of energetic forces, originating both from within and beyond the individual, which affects the form, direction, intensity, and duration of work-related behavior. This definition is sufficiently broad to yield an understanding of how such forces are behaving differently on different levels of conceptualization.

Throughout the review process, necessary changes were identified and made, and these changes were reported in the following section of the current paper.

### 4.1. Changes made throughout the process

Changes made to the search, inclusion, and analysis in the study were documented throughout the process. Firstly, the initially identified scope of the review entailed searching for, and including, documents from all fields of organizational research, psychology, and pedagogy, as these fields are prominent in motivation research. As the review unfolded, however, it became clear that the scope would have to be narrower, and so the review was restricted to documents from all fields of organizational research, education research, psychometric research, and all non-clinical fields of psychology and neuroscience. These research fields were selected based on a scoping study and a text-mining analysis of relevant studies, conducted with TerMine (Frantzi et al., 2000). Secondly, some keywords were not included in the initial protocol but were added as they emerged as central terms throughout the search process, these terms will be flagged with an asterisk in the following section. Thirdly, the main systematic search was carried out in December 2020, with results continuously updated with new publications until the beginning of November 2022 using Web of Science.

### 4.2. Inclusion and exclusion criteria

Papers from all years were included, as the number of studies published on cross-task motivation was quite limited. All documents in English or including an English abstract were included. All documents published in peer-review journals, as conference papers, or as accepted dissertations were included in initial screening. The initial inclusion of so called “gray literature” (evidence not published in commercial publication) was done in order to lessen the potential for publication bias. The papers included had a clear measure of motivation, distinguished clearly between tasks or situations, and contained a conceptualization of how motivation in one task or situation affect motivation in another. Different conceptualizations and angles were included in order to comply with the principle of pluralism (Wong et al., 2013). Only empirical papers were included, this included both qualitative and quantitative papers.

### 4.3. Search process

**Figure 1:**
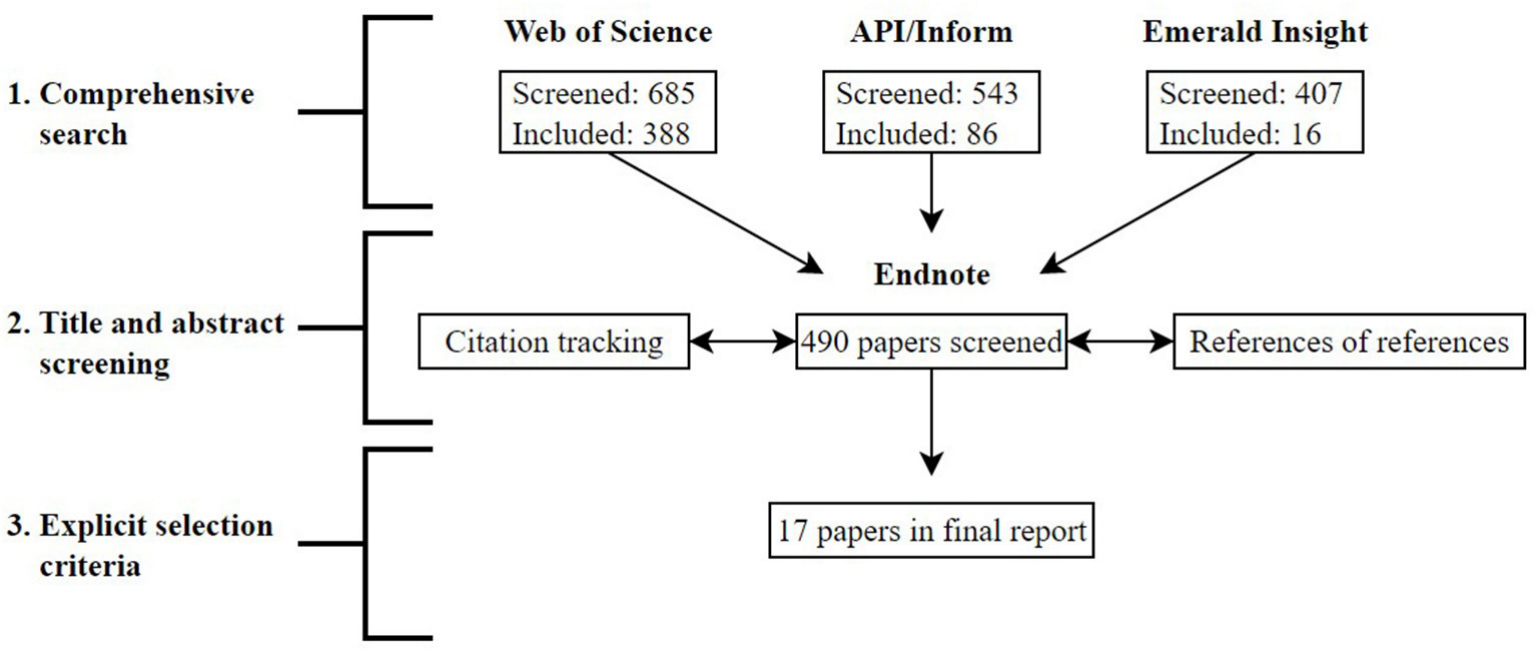
Flow chart of search.

Web of Science was used as principal search system, in accordance with Gusenbauer and Haddaway (2020), and API/Inform and Emerald Insight were included as additional search systems in order to ensure that all relevant documents were identified in searches. Search terms used in all three search systems were the terms motivation AND task OR assignment^*^ OR job OR situation AND dynamic OR cross OR change OR time OR interdependence^*^. Analysis of search results was conducted in three phases (see Figure 1 for a flow chart of search results). The first phase was the comprehensive search phase, where all articles with relevance to search terms were exported to Endnote X9 for further screening. The second phase was the title and abstract screening phase, where the title, abstracts, and keywords of all articles were screened for further inclusion. In cases where documents were not available as full texts, corresponding authors were contacted. The last phase was the explicit selection criteria phase, where all articles were read in full before final inclusion. Searches in all three search systems yielded 1,635 matches, of which the final included sample of documents consisted of 17 documents.

### 4.4. Data extraction and synthesis

In concordance with the meta-narrative method, included papers for review needed to be analyzed for meta-narratives, storylines within the literature, which could then be compared, contrasted, or combined in order to build theory (Greenhalgh et al., 2005). All papers were organized using Endnote X9, including meta-data and PDF copies. Papers were then read and analyzed, by means of thematic analysis. After an initial stage of familiarization and coding for recurring topics within, they were coded for meta-narratives using a combination of in-text tags, summaries, and extraction forms. This process was guided by the principle of pluralism from the meta-narrative methodology; illuminating the topics based on perspectives inherent to each research tradition (Greenhalgh et al., 2005). These meta-narratives were then systematically applied to all papers in order to synthesize across traditions, exploring commonalities and differences between meta-narratives and meta-theorizing.

## 5. Results

### 5.1. Characteristics of included papers

Although no restriction was put on publication year, 13 of the 17 articles were published after 2010, while one article was published in 2005, one in 1996, and the oldest article was published in 1991. Six of the articles were published in organizational journals, seven were published in journals of social, behavioral, and experimental psychology, three were published in neuroscience, and one article was published in educational research. All of the articles were quantitative in nature, with eight studies being experimental studies, three being naturalistic studies, and the remaining five combining experimental studies and naturalistic studies. For a detailed view of paper characteristics and their findings, see Table 1.

**Table 1:** Characteristics of papers included in review.

**References**	**Topic of journal**	**Main topic of interest**	**Main hypothesis**	**Support (yes/no)**	**Method**
Becker et al. (2019)	Psychology	Attention and conflict adaptation	Motivational cues in task 2 distract from motivational conflict in task 1	Yes	Experiment
Chadi et al. (2017)	Organizational science	Meaning of work	Loss of meaning decreases performance in a follow-up task	Yes	Field
Enzle et al. (1996)	Psychology	Basic need frustration	Autonomy and competence in task 1 yield higher intrinsic motivation in task 2	Yes	Experiment
Fang et al. (2017)	Psychology	Basic need frustration	Competence frustration in one course yields higher intrinsic motivation in a subsequent course	Mixed	Field
Fang et al. (2018)	Neuroscience	Basic need frustration	Competence frustration in task 1 yields higher motivation in task 2	Yes	Experiment
Fang et al. (2020)	Neuroscience	Basic need frustration	Autonomy frustration in task 1 yields higher autonomous motivation in task 2	No	Experiment
Fang et al. (2022)	Psychology	Basic need frustration	Autonomy frustration in task 1 yields higher intrinsic motivation in task 2	Yes	Experiment
Hogenelst et al. (2022)	Organizational science	Autonomous motivation	Measures of within-person variation in motivation over tasks reveal more nuanced processes than do between-person comparisons of motivation	Yes	Field
Meng and Ouyang (2020)	Psychology	Meaning of work	Loss of meaning in task 1 yields a higher supply of labor in task 2	Yes	Experiment
Newton et al. (2020)	Organizational science	Attention and engagement	Engagement in one task fosters engagement in a subsequent task	Yes	Field and experimental
Parker et al. (2013)	Organizational science	Basic need frustration	Non-self-determined participants have decreased intrinsic motivation over trials when task autonomy is high	Yes	Experiment
Philippe et al. (2019)	Organizational science	Memory and basic needs	Memories of need satisfaction yield higher self-determined motivation in a subsequent task	Yes	Field
Radel et al. (2014)	Educational research	Basic need frustration	Lack of autonomy in one task yields higher intrinsic motivation in a subsequent task	Yes	Field and experiment
Ratelle et al. (2005)	Psychology	Cued activation and motivation	Cue associated with controlling task yield lower intrinsic motivation subsequently	Yes	Experiment
Shin and Grant (2019)	Organizational science	Intrinsic motivation	Intrinsic motivation in task 1 reduces performance through boredom in task 2	Mixed	Field and experiment
Wei et al. (2020)	Neuroscience	Meaning of work	Loss of meaning in task 1 yields higher autonomous motivation in task 2	Yes	Experiment
Wong and Campion (1991)	Organizational science	Task and job motivation	The motivational value of jobs can be predicted from the motivational value of tasks, interdependence, and similarity	Yes	Field/mixed

### 5.2. Meta-narratives of cross-task motivation

With the 17 studies originating in different research traditions, with different topics of interest, the key variables such as motivation have been conceptualized in several ways across the studies. In the following section on results, differences in conceptualization and reasons for inclusion will be provided continuously. The following section outlines the four meta-narratives that ran through the studies with references to the papers that discussed the narratives. Not all papers discussed the different narratives, as the narratives emerged as a consequence of the fields of research and topics of interest of each paper.

#### 5.2.1. Restoration effects after need frustration

The most prominent meta-narrative of the papers was the topic of restoration effects after need frustration, which six of the papers investigated. All of these papers had their theoretical basis in Self-Determination Theory (SDT) (Deci and Ryan, 1985). The topic entails a lack of satisfaction of the basic psychological needs (autonomy, competence, and relatedness), which in the SDT are proposed to promote intrinsic motivation, which is thought to lead to a restoration process as the person seeks need satisfaction. This restoration process entails that the individual respond to the lack of need satisfaction by readjusting themselves so that subsequent motivation increases (Fiske, 2004; Veltkamp et al., 2009). Such a mechanism would explain cross-task effects of motivation where a lack of intrinsic motivation in one task would yield higher intrinsic motivation in a subsequent task. Of the six studies in the sample, three studies yielded results supporting the proposed relationship between prior need frustration and subsequent motivation. Fang et al. (2018) found that competence frustration in one task leads to an enhanced motivation to win in a subsequent task, measured using electroencephalogram (ERP), supporting the restoration hypothesis. Radel et al. (2014) found that students reported more intrinsic motivation for a music class when it was preceded by a controlling class, and in an experiment, participants who first learned to play a game in a controlling context had more interest in a second game. Fang et al. (2022) added further support to the autonomy restoration hypothesis, showing that autonomy-deprived individuals are more intrinsically motivated to work in a subsequent task that provides them the opportunity to regain autonomy, than individuals that are not autonomy deprived.

Two studies yielded opposite results, however, with Fang et al. (2020) finding that participants who experienced autonomy frustration in a task setting, have decreased motivation in the subsequent task setting, compared to the control group. Parker et al. (2013) found that intrinsic motivation of non-self-determined, but not of self-determined, individuals decreased continuously over settings where work control (autonomy) was high. In the study, Parker et al. (2013) described non-self-determined individuals as already experiencing autonomy frustrations, and so both these studies seem to suggest that autonomy frustration yields subsequent lower intrinsic motivation. Although several factors could be contributing to these conflicting findings, Fang et al. (2020) suggest that the reason may be that autonomy frustration can only give rise to restoration processes if the subsequent task is seen as competence-supporting (in other words not too difficult to master). It may then be the case that in the Fang et al. (2020) article, the second task was too difficult to support restoration and that this too was the case in the Parker et al. (2013) study.

One last study, nuancing the picture further, is another study by Fang et al. (2017). In this study, there was a U-shaped relationship between competence frustration in a preceding course and intrinsic motivation in a subsequent course. This U-shaped relationship entailed that up to a point, competence frustration was not affecting subsequent intrinsic motivation, but after that point, the two variables became positively correlated. One possible explanation for this finding is a proposed difference between low need satisfaction and need frustration (Vansteenkiste and Ryan, 2013). According to this view, low need satisfaction is not enough to initiate the same mechanisms as need frustration, which is a stronger sense of lack of basic needs. This could entail that need dissatisfaction give rise to a restoration effect only when the impact is large enough.

#### 5.2.2. Intrinsic and extrinsic motivation

The second meta-narrative from the sample was a more general investigation of intrinsic and extrinsic motivation. Enzle et al. (1996) found that both autonomy and competence in one activity caused more intrinsic motivation in a subsequent activity. By including expected and unexpected rewards in the subsequent activity, they also found that autonomy in the first activity decreased the negative effect of extrinsic reward on intrinsic motivation in the subsequent activity. Shin and Grant (2019), the second study contributing to this narrative, found a U-shaped relationship between intrinsic motivation in a task and performance, explained by boredom, in a subsequent task. The proposed explanation for this U-shaped relationship is that for moderate levels of intrinsic motivation, there is a carryover effect so that moderate intrinsic motivation carries over to a subsequent activity, but for high levels of intrinsic motivation, a contrasting effect occurs. Such a contrast effect entails a comparison between two conditions, where the second condition is markedly different from the first, and the effect of this condition is that the difference between conditions is exaggerated (Zentall, 2005). In the case of the study by Shin and Grant (2019), the contrasting effect causes the subsequent activity to feel less interesting because the previous task was too interesting. The results from the study by Shin and Grant (2019) were replicated to some extent in the final study of this meta-narrative, the study by Hogenelst et al. (2022). In their study, they found that contrary to studies investigating motivation between persons on the job level, motivation measured multiple times at the level of within-person on the task level was not associated with same-day productivity and job satisfaction. Their explanation for this finding is in line with the finding of Shin and Grant (2019), as they hypothesize that individuals may have picked the most interesting tasks at the beginning of the day, reporting their motivation for these, and then followed up with less and less interesting tasks throughout the day, before reporting their productivity and job satisfaction at the end of the day. This would yield relatively high average motivation across the day, but then job satisfaction and productivity scores at the end of the day would be influenced by the last reported motivation scores, and motivation for tasks throughout the day would be subject to contrasting effects, as the individuals performed the most interesting tasks to begin with.

#### 5.2.3. Cognitive carryover effects

Another meta-narrative among the identified studies was the topic of cognitive carryover effects. Here, the focus of the studies was on the cognitive aspects of cross-task motivation, such as memory (Philippe et al., 2019) and attention (Ratelle et al., 2005; Becker et al., 2019; Newton et al., 2020). The study by Philippe et al. (2019) found that memories of need satisfaction predicted increased self-determined motivation in a subsequent task, which supports a carryover effect of motivation that is sustained by memory processes. Ratelle et al. (2005), Becker et al. (2019), and Newton et al. (2020) all looked at attention in relation to cross-task motivation. Newton et al. (2020) utilized engagement as a “motivational state” and found that engagement in one task carries over to a subsequent task, but engagement also leads to attention residue that impedes subsequent engagement to a certain degree. Becker et al. (2019) made similar findings in the opposite direction, finding that *conflict adaptation effect* resulting from motivational conflict in a preliminary task, was reduced when motivational distractors were introduced in a subsequent task. The last study within this narrative showed that being presented with a tone in an experiment that was previously associated with a controlling task, undermined the intrinsic motivation of participants (Ratelle et al., 2005). Taken together, these four studies indicate that cognitive abilities such as memory and attention play a central role in shifts in motivation.

#### 5.2.4. Meaning of work

The fourth meta-narrative appearing from the identified studies was the topic of disappearance of the meaning of work, with meaningfulness being compared to identified regulation (a type of extrinsic motivation) in SDT (Meng and Ouyang, 2020). Three studies all examined the effect of a sudden loss of meaning in one task on motivation in a subsequent task. Meng and Ouyang (2020) found that participants that suffered the loss of meaningfulness in a task worked more than controls in a subsequent task, while Wei et al. (2020) found that participants who experienced a sudden loss of meaning in a task had higher autonomous motivation for a subsequent task. Both of these studies explain their finding with the fluid compensation hypothesis from the Meaning Maintenance Model (Heine et al., 2006). This hypothesis is quite similar to the restoration hypothesis, stating that loss of meaning in a task may elicit a response where the individual seeks to find meaning in another event/task, increasing their motivation for that task (Heine et al., 2006). The last study of the three investigated the sudden loss of meaning in a naturalistic setting and found that sudden loss of meaning yielded decreased motivation in a follow-up task (Chadi et al., 2017), seemingly contradicting the other two studies. Wei et al. (2020), however, propose that the different findings may be due to participants in the study by Chadi et al. experiencing the initial task and the subsequent task as related to each other and similar, and therefore do not experience the second task as an independent arena for regaining meaning (Wei et al., 2020). This potential explanation is an interesting one if it is the case that task similarity or interdependence affects cross-task motivational effects.

#### 5.2.5. Task motivation, similarity, and interdependence

One study did not align with any of the existing meta-narratives but is still central to the topic of cross-task motivation. Wong and Campion (1991) tested a model of motivational job design where the motivational value of jobs was predicted from the motivational value of tasks, task interdependence, and task similarity on 67 different jobs. They found that increased motivational value of tasks and increased task interdependence led to higher motivational value of jobs. Task similarity did not have an effect. This is an interesting point concerning the previously mentioned possibility that task interdependence and similarity may affect cross-task motivational processes, such as in the study of loss of meaning (Chadi et al., 2017).

## 6. Discussion

### 6.1. Synthesis of the findings

Although four different meta-narratives frame the literature on cross-task motivation, there are still several commonalities between the 17 studies. All studies enlighten different ways motivation in one task may affect motivation in another, depending on the type, strength, and context of motivation. Taken together, results suggest that lack of intrinsic motivation through need frustration results in increased intrinsic motivation in a subsequent task if the need frustration is substantial enough (Radel et al., 2014; Fang et al., 2017, 2018, 2022), although not if the subsequent task is too difficult to support a restoration process (Fang et al., 2020). A similar effect exists for the sudden disappearance of meaning, where the loss of meaning in one task can cause increased motivation in a subsequent task (Meng and Ouyang, 2020; Wei et al., 2020), but not if the subsequent task is too similar or connected to the first to support a compensation (Chadi et al., 2017). Further, high intrinsic motivation in one task will carry over to another task (Enzle et al., 1996; Philippe et al., 2019; Newton et al., 2020), but not if the contrast in degree of interest between the first and the subsequent task is so large that a psychological contrast effect takes place (Shin and Grant, 2019; Hogenelst et al., 2022).

### 6.2. A meta-theoretical framework for understanding cross-task motivation

**Figure 2:**
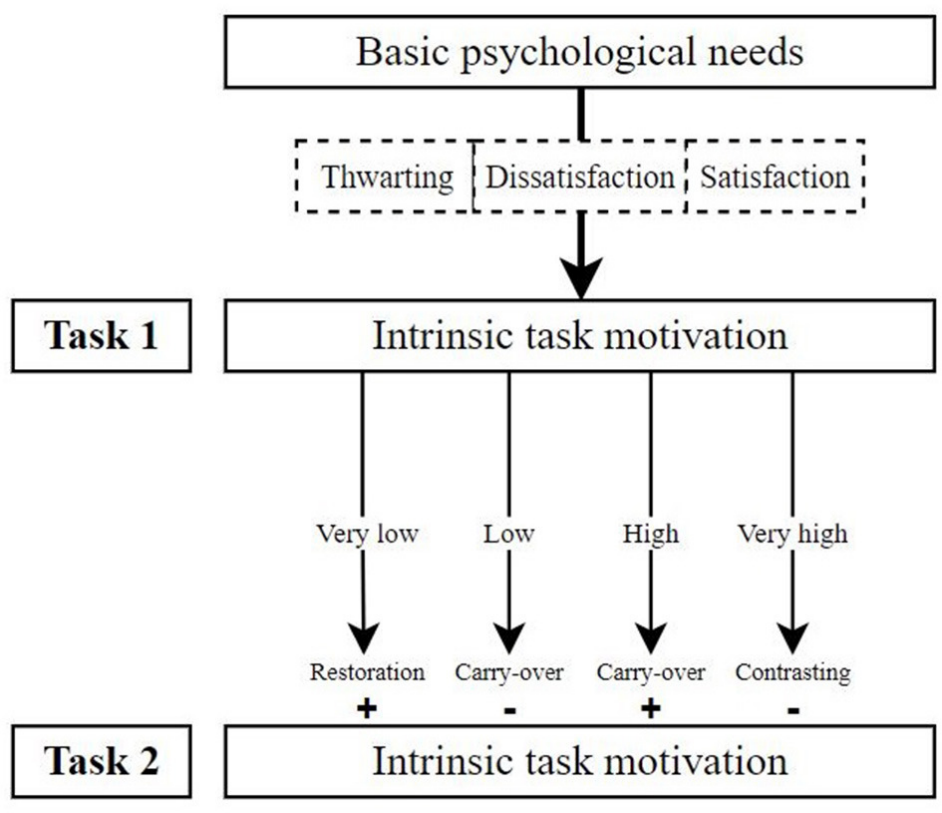
A synthesized model of cross-task motivation.

In light of these findings, a possible meta-theoretical framework for understanding cross-task motivation emerges. Expanding upon the hierarchical model of intrinsic and extrinsic motivation by Vallerand (1997, 2001), this framework outlines how motivation in one task may carry over to a subsequent task, affecting subsequent task motivation depending on the magnitude of the previous motivational experience. If there has been a need frustration in a previous task, intrinsic motivation may increase for the subsequent task due to restoration effects if the need frustration was grave enough or intrinsic motivation for the subsequent task decrease due to carry-over effects if the need frustration was less impactful. With high intrinsic motivation in an initial task, subsequent motivation is either high due to carryover effects or lowered due to psychological contrasting. Several factors seem to influence the magnitude of the motivational experiences, such as task similarity, task interdependence, and different need satisfaction of different needs. Figure 2 shows an illustration of the synthesized model, where basic psychological need fulfillment predicts intrinsic situational motivation for a task in line with the hierarchical model by Vallerand (1997), and this task motivation affects subsequent motivation in a second task.

### 6.3. Conclusion and implications for practice

The current meta-narrative review has analyzed and synthesized the existing literature on cross-task motivation. Seventeen studies were identified as contributing to the topic, and four meta-narratives were identified from these studies. Based on these findings, a meta-theoretical framework for understanding different kinds of cross-task motivational effects was proposed, where the magnitude and type of initial motivational experiences affected subsequent task motivation. This proposed framework is an extension of existing theories of motivation, both self-determination theory (Deci and Ryan, 1985) and the hierarchical model of intrinsic and extrinsic motivation (Vallerand, 1997). This, as existing theoretical frameworks, does not account for how motivation changes between tasks or how existing motivation may affect subsequent motivation.

With employees working in increasingly complex jobs consisting of multiple tasks and frequent task switching, possessing knowledge about how different sequences of tasks with different motivational properties may affect employee motivation is valuable in order to ensure positive motivational outcomes such as employee wellbeing and performance. This implication for practice is future supported by the fact that several of the motivational processes identified are paradoxical, and thereby hard to foresee without necessary empirical evidence. The fact that need frustration may actually enhance motivation in a subsequent task (Radel et al., 2014; Fang et al., 2018, 2022), or that too high intrinsic motivation in one task may actually diminish motivation in a subsequent task (Shin and Grant, 2019) is information useful to practitioners as it is not self-evident. In addition to informing practitioners within the work field, this model may also inform practitioners and researchers in other areas, such as in developing surveys and experiments with multiple tasks for participants to complete, so that effects on motivation may be controlled.

### 6.4. Limitations

One limitation of the current study results from the utilization of a meta-narrative review methodology. In meta-narrative reviews, the guiding principle of pragmatism entails that what to include in the review must be guided by what is most useful (Wong et al., 2013). In this study, four meta-narratives were distilled from the 17 studies as they were judged the most illuminating of the topic. However, all 17 studies included interesting additional variables and theoretical contributions that did not make it into the current review, which may have contributed to another narrative, had they been included.

A second limitation entails the methodology of the systematic search. Although a vast number of articles were screened, choices were made both in choosing the appropriate keywords, choice of research domains, and search systems, meaning that there is always a possibility that some studies were missed in the search process. However, as search terms were meticulously chosen and a large number of identified documents were screened, it is reasonable to believe that the systematic search was accurate in identifying the relevant studies.

A last limitation of the current review is that with such syntheses of existing research, the limitations of the included studies become possible limitations of the current one. Limitations to the studies include the use of self-report to measure motivation in several studies, although three studies utilized ERP to assess motivation (Fang et al., 2018, 2020; Wei et al., 2020), and several measures of motivation that are not usually utilized, e.g., supply of labor (Meng and Ouyang, 2020) or engagement as a motivational state (Newton et al., 2020). Of 17 studies, only six included some form of field data, which is a possible limitation to the ecological validity of the findings.

### 6.5. Future directions

Future research on cross-task motivation should test the proposed model of different mechanisms of cross-task motivation. The research that has been conducted up until this point has focused mainly on intrinsic motivation, and so studies looking at other types of motivation, for example extrinsic motivation, could contribute new information to a quite sparsely researched field.

The current study identified no qualitative studies investigating cross-task motivation, and so a great potential avenue for understanding the phenomenon of fluctuating motivation would be through theoretical development from such studies. Future studies should also seek to investigate the proposed role of different levels of motivation in cross-task motivational processes, in line with the mechanisms proposed in the hierarchical model of intrinsic and extrinsic motivation.

Furthering a holistic understanding of cross-motivational processes could be achieved by future studies aligning the current model of cross-task motivation with behavioral and attitudinal frameworks for understanding motivated behavior, self-regulation and decision making, such as the literature on aspiration levels, self-efficacy, and ego-depletion. This would be a fruitful avenue for identifying additional mechanisms through which motivation fluctuates over time.

## Data availability statement

The original contributions presented in the study are included in the article/supplementary material, further inquiries can be directed to the corresponding author.

## Author contributions

The author confirms being the sole contributor of this work and has approved it for publication.
